# Cell‐free DNA is elevated in the serum of patients with hidradenitis suppurativa

**DOI:** 10.1111/1346-8138.16676

**Published:** 2022-12-14

**Authors:** Daniel G. W. Johnston, Roisin Hambly, Niamh Kearney, Desmond J. Tobin, Brian Kirby

**Affiliations:** ^1^ Discipline of Anatomy, School of Medicine Trinity Biomedical Sciences Institute, Trinity College Dublin Dublin Ireland; ^2^ UCD Charles Institute of Dermatology University College Dublin Dublin Ireland; ^3^ Charles Centre for Dermatology St Vincent's University Hospital Dublin Ireland; ^4^ UCD Conway Institute, University College Dublin Dublin Ireland

**Keywords:** cell‐free DNA, hair follicle, hidradenitis suppurativa, skin, systemic inflammation

1

Hidradenitis suppurativa (HS) is a chronic inflammatory skin disease characterized by recurring inflammatory lesions in intertriginous areas that result from the rupture of occluded hair follicles. The etiology of this disease is poorly understood, but it is increasingly apparent that numerous immunological pathways are dysregulated, with elevated numbers of infiltrating immune cells and cytokines present both locally and systemically.^1^ In recent years, several studies have implicated the innate immune system in HS pathogenesis, with macrophages and neutrophils recognized as key players in different phases of the disease. Neutrophils have been shown to be a predominant infiltrating leukocyte in HS lesions, and it has been demonstrated that on entering the site of inflammation they disgorge their DNA‐laden chromatin in the form of neutrophil extracellular traps (NETs).^2^ NETs are a means of innate immune defense against pathogens but have also been implicated in autoinflammatory diseases when aberrantly deployed, leading to the formation of pathogenic citrillunated autoantibodies as is the case in HS.^2^ Given that there is an increase in the amount of local cell‐free DNA, a known damage‐associated molecular pattern (DAMP), we wished to know if this was mirrored systemically as circulating DNA. Circulating DNA is a known biomarker of inflammatory diseases, such as systemic lupus erythematosus (SLE).^3^

We measured cell‐free DNA in the sera of 20 HS patients and compared it to 12 healthy people acting as a control group (Figure 1a). All HS patients bar one had moderate to severe disease, Hurley stages 2 (*n* = 17) and 3 (*n* = 2). The study was approved by the Medical Research Committee of St. Vincent's University Hospital. Cell‐free DNA was measured using a plate‐based SYBR Gold method described by Goldschtein et al., where DNA chelation to the fluorescent dye was detected using a SpectraMax M3 plate reader with Softmax Pro analysis software (Molecular Devices).^3^ Statistical analysis of the difference between the mean cell‐free DNA levels of the control group and the HS group was performed using GraphPad Prism (GraphPad Software) and an unpaired Student's *t*‐test with significance of *P* < 0.05. Correlation with clinical parameters C‐reactive protein (CRP), age and body mass index (BMI) was done using Pearson's correlation coefficient.

**FIGURE 1:**
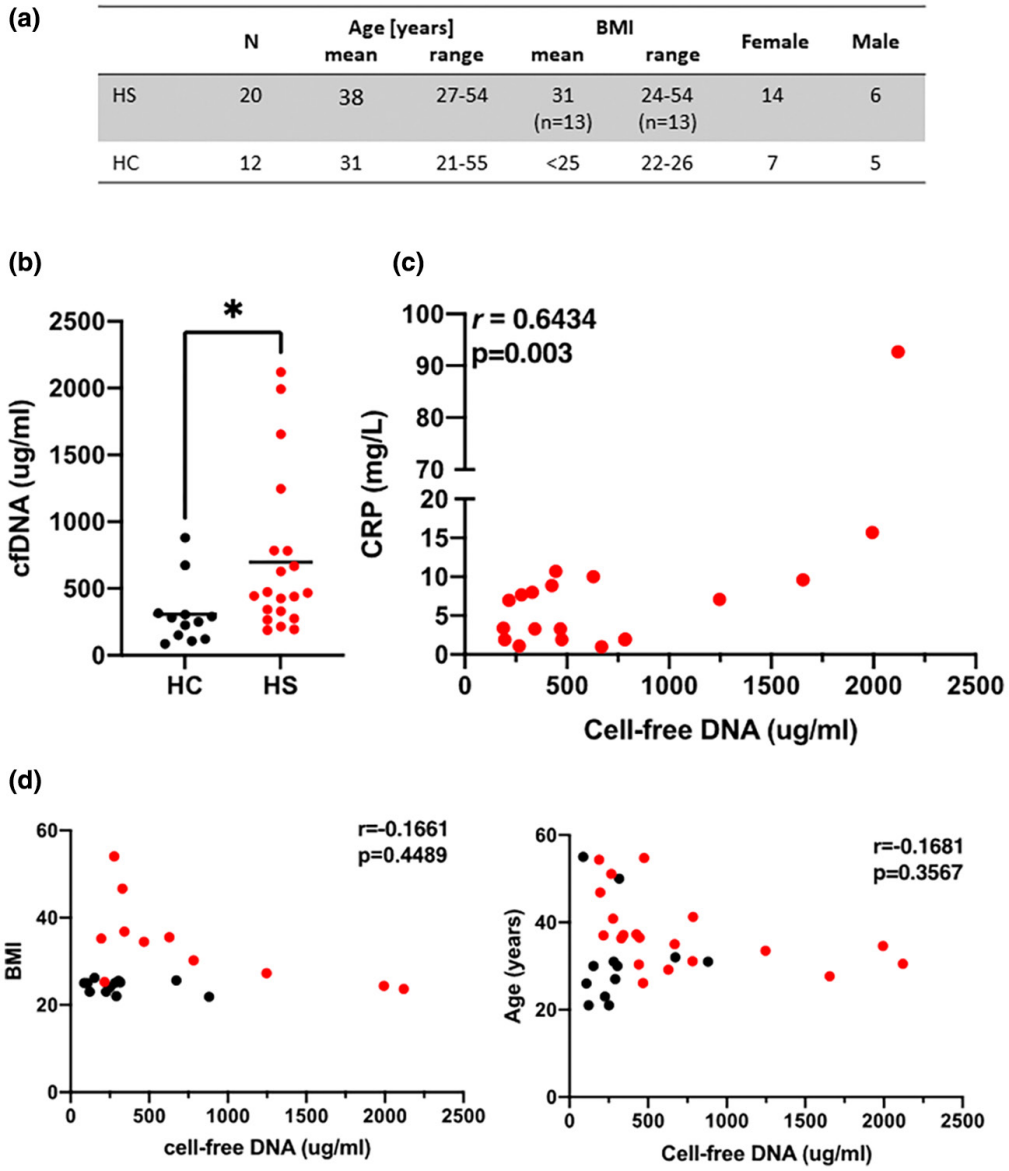
Patients with hidradenitis suppurativa (HS) have elevated levels of cell‐free DNA in serum. (a) Demographics of individuals with HS and healthy controls. Serum levels of cell‐free DNA were analyzed in HS (*n* = 20) and healthy control groups (*n* = 12). (b) Dot plot representing individual samples (HS = red, HC = black) with mean levels of cell‐free DNA (black line) in each group. *P* < 0.05 using unpaired Student's *t*‐test. (c, d) Scatter plots correlating cell‐free DNA with CRP levels, age and BMI. *P* < 0.05 using Pearson's correlation coefficient.

The mean serum level of cell‐free DNA was increased significantly in patients with HS (697.4 μg/ml ± 590.2, *P* < 0.0379) in comparison with healthy lean controls (307.8 μg/ml ± 237.6) (Figure 1b). Cell‐free DNA levels positively correlated with CRP (Figure 1c), a disease marker in HS.^1^ Cell‐free DNA did not correlate with age or BMI (Figure 1d).

To our knowledge, this is the first study to assess the concentration of cell‐free DNA in the serum of HS patients. Cell‐free DNA is mainly associated with cell death, a phenomenon understudied in HS apart from NETosis, as previously mentioned. It is a well‐known biomarker for rheumatic diseases such as rheumatoid arthritis and SLE, where it was first discovered to be present in excess in the 1960s.^4^ It has more recently become apparent that cell‐free DNA is present in excess in a wide variety of inflammatory disorders, including those at barrier sites, such as inflammatory bowel disease, coeliac disease, Sjogren's syndrome, and other diseases with an inflammatory component such as type 1 diabetes mellitus.^5^

Cell‐free DNA has also been shown to be dysregulated in the inflammatory skin disease psoriasis.^6^ In addition to alterations in the total amount present in the circulation, cell‐free DNA from different patients may have altered immunoregulatory properties. While ordinarily cell‐free DNA is not immunogenic due to its rapid degradation, when complexed with carrier molecules it can reach intracellular DNA‐sensing pattern recognition receptors (PRRs) of the innate immune system and lead to heightened inflammatory signaling. The immunogenicity of systemic cell‐free DNA is potentially relevant in HS where the role of PRRs is understudied and potential carrier molecules such as anti‐dsDNA autoantibodies and the antimicrobial peptide LL‐37 are present in excess.^1^ It has recently been suggested that the innate immune cytosolic DNA sensing STING pathway, controlled by the PRR IFI16, may be responsible for hair follicle hyperkeratinization and collapse.^1^, ^7^ This among the first reported circulating PRR‐triggering DAMP associated with HS. It suggests a link between aberrant cell death and innate immune activation through PRRs, which should be further explored in subsequent studies. This study is limited as the majority of patients had Hurley stage 2 disease. It would be interesting to explore the potential of cell‐free DNA as a disease severity‐specific HS biomarker in a larger study that includes dynamic severity scoring systems such as international hidradenitis suppurativa severity scoring system.

## 2 FUNDING INFORMATION

None to declare.

## 3 CONFLICT OF INTEREST

None to declare.
